# P-1336. Building Connections: Rise in Construction Projects correlated with New Cluster of Infantile Botulism in North Texas

**DOI:** 10.1093/ofid/ofae631.1513

**Published:** 2025-01-29

**Authors:** Amanda S Evans, William Wood

**Affiliations:** UT Southwestern Medical Center, Dallas, Texas; University of Texas Southwestern, Dallas, Texas

## Abstract

**Background:**

Regional clustering of infantile botulism is well documented in throughout the United States. We report a new cluster of cases in Northeast Texas.

**Figure:**
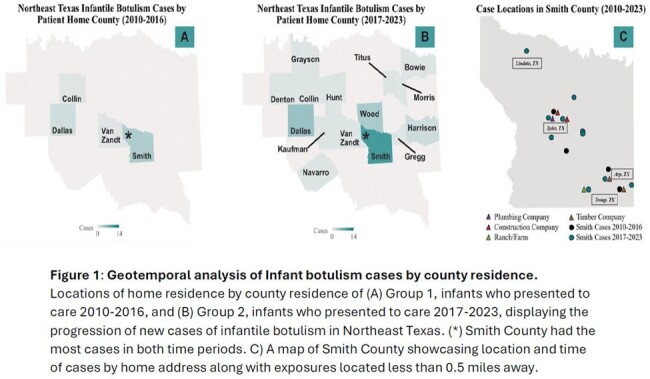

**Methods:**

We performed a single center retrospective chart review of all patients with suspected and confirmed infant botulism admitted to Children’s Medical Center Dallas from January 1, 2010 through December 31, 2023. Environmental conditions near each patient’s home prior to hospitalization were analyzed. County level data on annual birth, population, and death rates was collected from The United States Census Bureau. Construction permit data from Tyler, Texas was analyzed.

**Figure:**
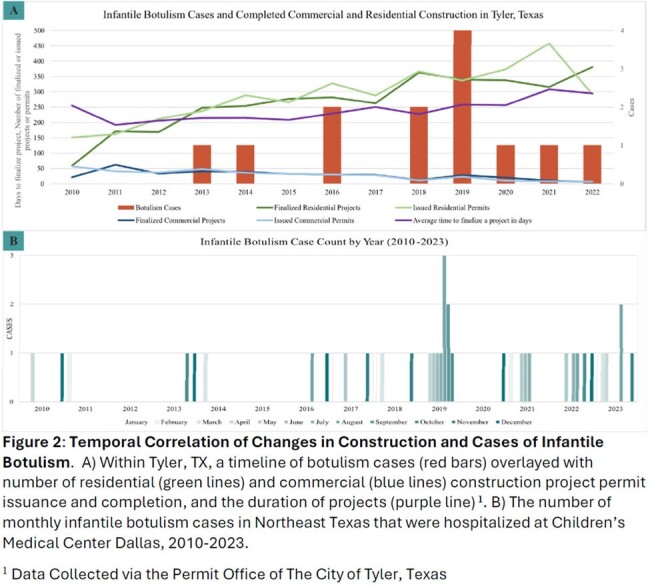

**Results:**

46 infants with suspected infant botulism presented to care and received botulism immune globulin from 2010-2023. 3 infants were excluded due to alternative neurologic diagnosis. Features of cohort (N=43) without alternative diagnosis included in Table 1. 39 infants without honey exposure were included in environmental analysis: Group 1: 2010-2016 (N=8 infants), and Group 2: 2017-2023 (N=31 infants). The mean number of yearly residential construction permits issued (235.0 vs 352.7, p=0.01) and projects finalized (208.6 vs 333.5, p=0.01) increased from 2010-2016 to 2017-2022. The mean number of yearly commercial construction permits issued (40.1.0 vs 14.5, p=< 0.01) and projects finalized (36.9 vs 17.5, p=0.01) decreased between 2010-2016 as compared to 2017-2022. A sudden cessation of infantile botulism cases occurred during the COVID-19 pandemic that appears to coincide with delays in construction projects being complete. There were no significant differences between the 2 cohorts in environmental changes near the patient’s home address: temperature, precipitation, wind speed or direction, soil moisture, or drought index (Table 2). Specific humidity was significantly higher (p=0.01) in the 30-60 days before admission for patients presenting 2017-2023 as compared to 2010-2016 (-0.53 g/kg vs 0.70 g/kg).

**Figure:**
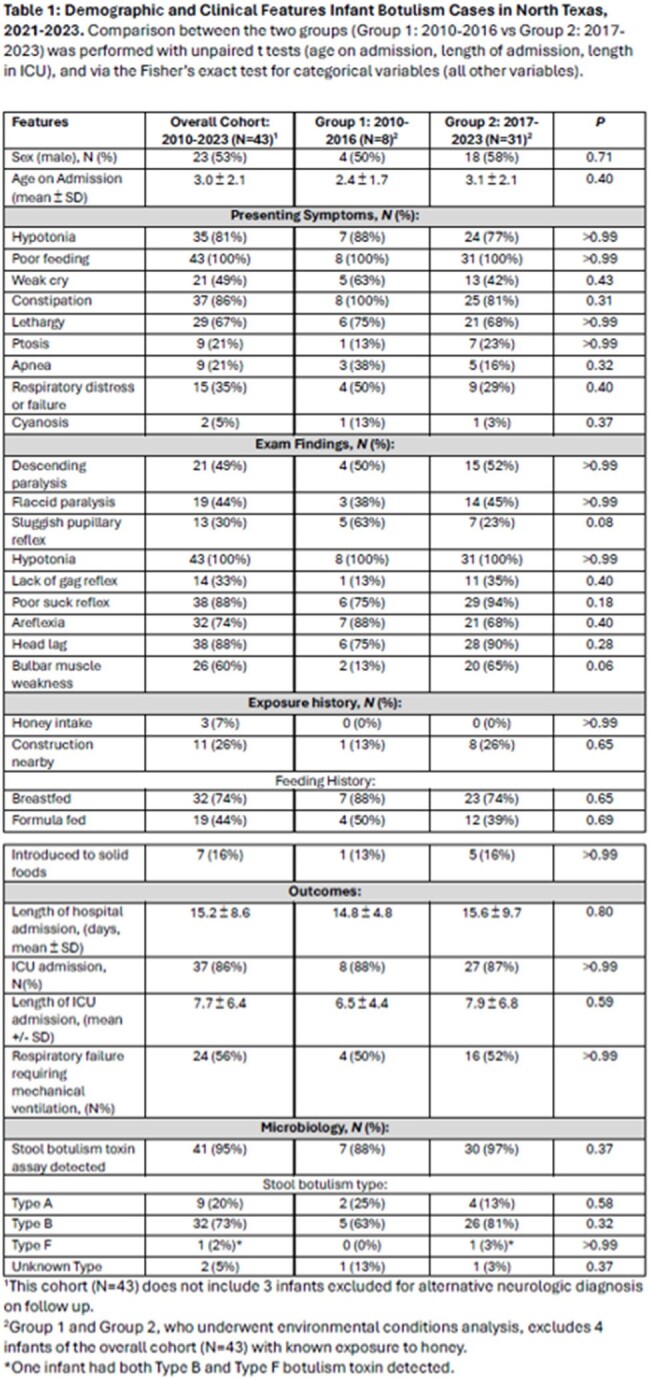

**Conclusion:**

Changes in local construction patterns are associated with a notable increase in infantile botulism cases in Northeast Texas over the past 14 years. Changes in environmental conditions do not appear to play a large role in this increasing case count.

**Figure:**
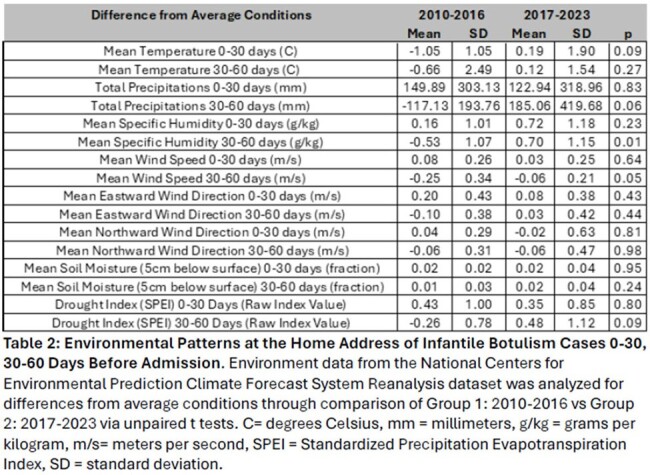

**Disclosures:**

**All Authors**: No reported disclosures

